# The Story of 5d Metallocorroles: From Metal–Ligand Misfits to New Building Blocks for Cancer Phototherapeutics

**DOI:** 10.1021/acs.accounts.1c00290

**Published:** 2021-07-23

**Authors:** Abraham B. Alemayehu, Kolle E. Thomas, Rune F. Einrem, Abhik Ghosh

**Affiliations:** Department of Chemistry, UiT—The Arctic University of Norway, N-9037 Tromso, Norway

## Abstract

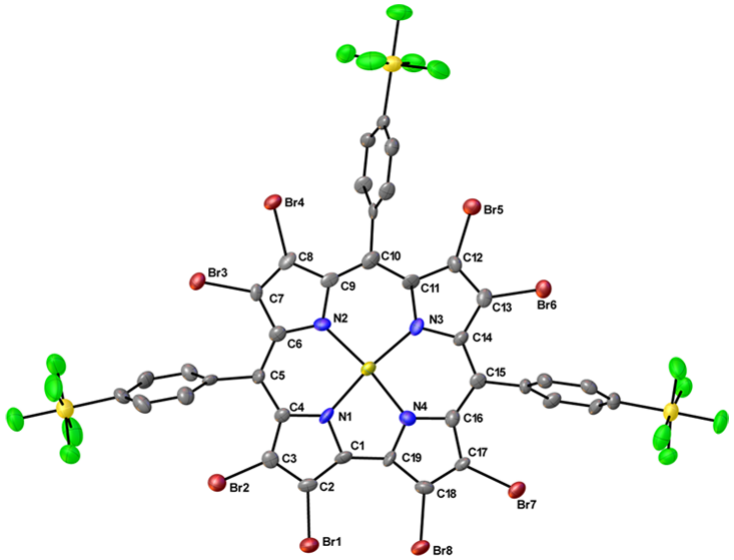

Porphyrin chemistry is Shakespearean: over a century of study has not withered the field’s apparently infinite variety. Heme proteins continually astonish us with novel molecular mechanisms, while new porphyrin analogues bowl us over with unprecedented optical, electronic, and metal-binding properties. Within the latter domain, corroles occupy a special place, exhibiting a unique and rich coordination chemistry. The 5d metallocorroles are arguably the icing on that cake.

New Zealand chemist Penny Brothers has used the word “misfit” to describe the interactions of boron, a small atom with a predilection for tetrahedral coordination, and porphyrins, classic square-planar ligands. Steve Jobs lionized misfits as those who see things differently and push humanity forward. Both perspectives have inspired us. The 5d metallocorroles are misfits in that they encapsulate a large 5d transition metal ion within the tight cavity of a contracted porphyrin ligand.

Given the steric mismatch inherent in their structures, the syntheses of *some* 5d metallocorroles are understandably capricious, proceeding under highly specific conditions and affording poor yields. Three broad approaches may be distinguished.

(a) In the *metal–alkyl approach*, a free-base corrole is exposed to an alkyllithium and the resulting lithio-corrole is treated with an early transition metal chloride; a variant of the method eschews alkyllithium and deploys a transition metal–alkyl instead, resulting in elimination of the alkyl group as an alkane and insertion of the metal into the corrole. This approach is useful for inserting transition metals from groups 4, 5, and, to some extent, 6, as well as lanthanides and actinides.

(b) In our laboratory, we have often deployed a *low-valent organometallic approach* for the middle transition elements (groups 6, 7, 8, and 9). The reagents are low-valent metal–carbonyl or −olefin complexes, which lose one or more carbon ligands at high temperature, affording coordinatively unsaturated, sticky metal fragments that are trapped by the corrole nitrogens.

(c) Finally, a *metal acetate approach* provides the method of choice for gold and platinum insertion (groups 10 and 11).

This *Account* provides a first-hand perspective of the three approaches, focusing on the last two, which were largely developed in our laboratory. In general, the products were characterized with X-ray crystallography, electrochemistry, and a variety of spectroscopic methods. The physicochemical data, supplemented by relativistic DFT calculations, have provided fascinating insights into periodic trends and relativistic effects.

An unexpected feature of many 5d metallocorroles, given their misfit character, is their remarkable stability under thermal, chemical, and photochemical stimulation. Many of them also exhibit long triplet lifetimes on the order of 100 μs and effectively sensitize singlet oxygen formation. Many exhibit phosphorescence in the near-infrared under ambient conditions. Furthermore, water-soluble ReO and Au corroles exhibit impressive photocytotoxicity against multiple cancer cell lines, promising potential applications as cancer phototherapeutics. We thus envision a bright future for the compounds as rugged building blocks for new generations of therapeutic and diagnostic (theranostic) agents.

## Key References

- ThomasK. E.; AlemayehuA. B.; ConradieJ.; BeaversC.; GhoshA.Synthesis and Molecular Structure of Gold Triarylcorroles. Inorg. Chem.2011, 50, 12844–128512211160010.1021/ic202023r.^1^ Not the first paper to report gold corroles, but the first to make them readily accessible. The “acetate method” described herein is still the method of choice for Au corroles.
- AlemayehuA. B.; Vazquez-LimaH.; BeaversC. M.; GagnonK. J.; BendixJ.; GhoshA.Platinum Corroles. Chem. Commun.2014, 50, 11093–1109610.1039/c4cc02548b24911328.^2^ Perhaps the best illustration of the sheer capriciousness of 5d metal insertion into corroles and of the ultimate success of the acetate method.
- AlemayehuA. B.; GagnonK. J.; TernerJ.; GhoshA.Oxidative Metalation as a Route to Size-Mismatched Macrocyclic Complexes: Osmium Corroles. Angew. Chem., Int. Ed.2014, 53, 14411–1441410.1002/anie.20140589025346094.^3^ One of the simpler, higher-yielding syntheses for 5d metallocorroles.
- EinremR. F.; GagnonK. J.; AlemayehuA. B.; GhoshA.Metal-Ligand Misfits: Facile Access to Rhenium-Oxo Corroles by Oxidative Metalation. Chem. - Eur. J.2016, 22, 517–5202663995110.1002/chem.201504307.^4^ Arguably the simplest among all syntheses of 5d metallocorroles.

## 1 Introduction

**Chart 1:**
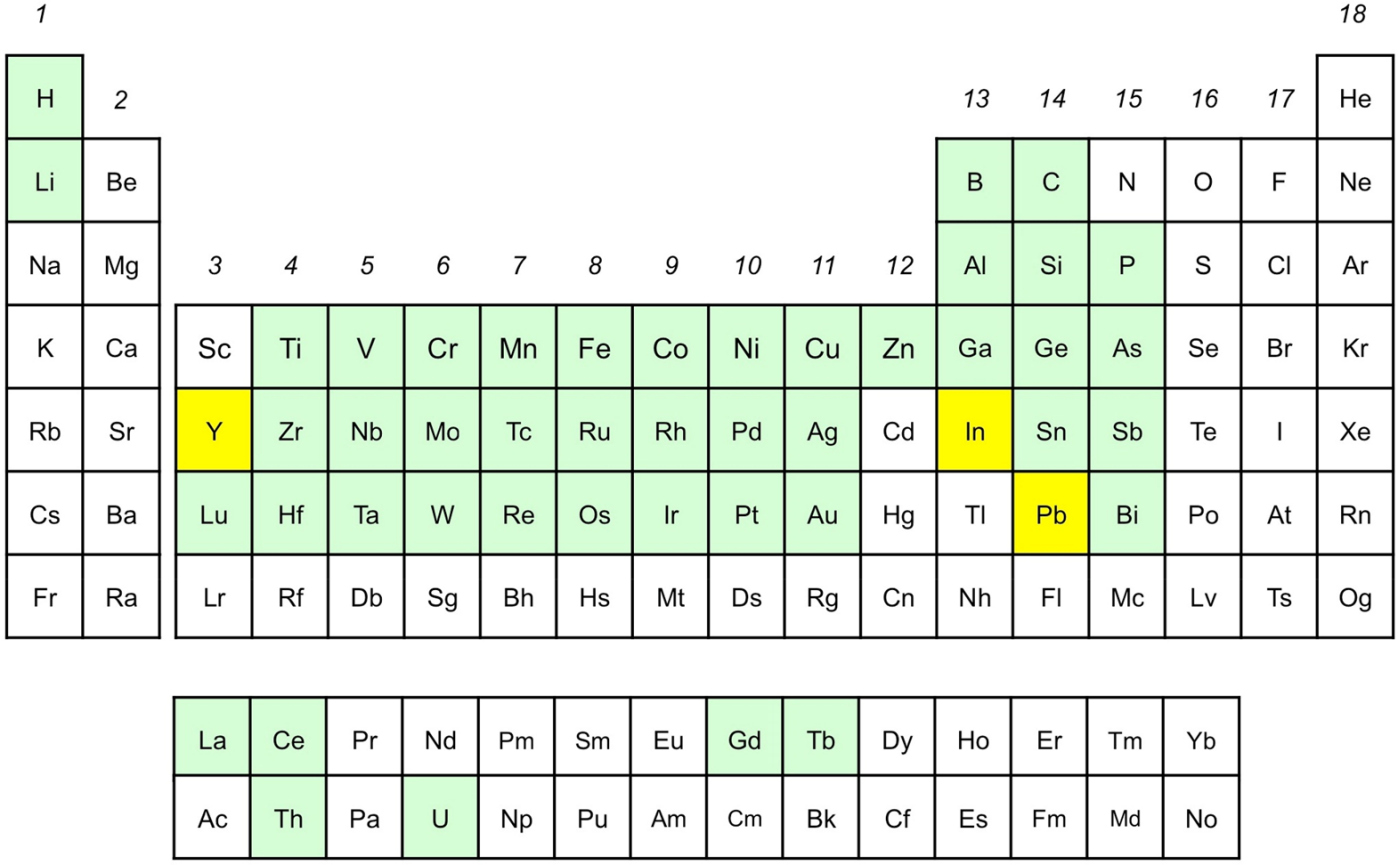
Periodic Table of Corrolesa

Over the last dozen or so years, an unlikely new chapter has been added to the coordination chemistry of porphyrin-type molecules, namely, the synthesis and characterization of the 5d metallocorroles. Unlikely—because the sterically constrained N_4_ cores of corroles are snug even for first-row transition metals, so until a few years ago it was far from clear that 5d transition metals would eventually all yield stable complexes.^5,6^ One such complex, Re^V^O *meso*-tris(trifluoromethyl)corrole, Re^V^[TCF_3_C](O), however, had already been reported in 1998, as the product of an unexpected ring contraction during an attempted rhenium insertion into a porphyrin.^7^ A full decade elapsed until the next 5d metallocorroles, six-coordinate iridium(III) corroles, were synthesized by Gray and co-workers.^8^ Gold corroles were the next “obvious” target and were synthesized essentially simultaneously by us and by Gross’s lab at the Technion.^9,10^ Meanwhile, Arnold and co-workers at the University of California, Berkeley, developed the chemistry of early transition metal corroles, focusing on groups 4 and 5.^11^ In 2014, the two remaining dominoes, platinum^2^ and osmium^3^ corroles, fell at our Tromsø laboratory, “completing” the 5d series and indeed an entire rectangle of transition metals from group 4 through 11 in the periodic table of corroles (Chart 1). This Account presents the coordination chemistry of 5d metallocorroles, largely as it unfolded in our laboratory, focusing on synthetic methods, structural chemistry, optical, electrochemical, and photophysical properties, and applications to photomedicine, particularly photodynamic therapy.

a Green backgrounds indicate structurally characterized corroles; yellow backgrounds indicate known derivatives that have not been structurally characterized.

**Figure 1:**
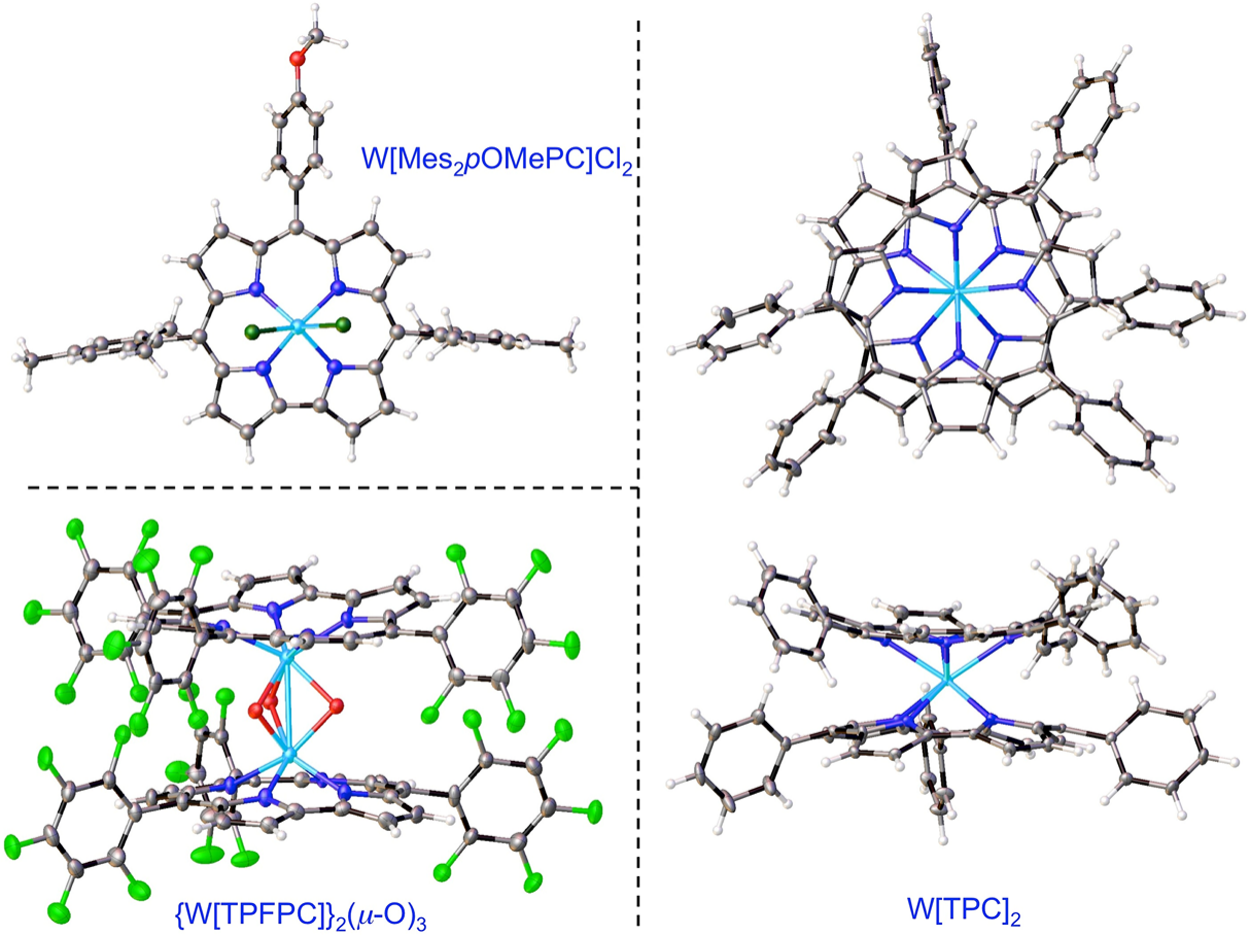
X-ray structures of selected W corroles. See Table 1 for key structural data: CAWVAZ, WUNZUC, and OKIJID.

The synthetic methods available for 4d and 5d metallocorroles are more limited relative to those available for 3d metallocorroles as well as for metalloporphyrins, reflecting the steric mismatch between the large size of the heavier transition metal ions and the corroles’ tight central cavities. (The natural radius of the corroles’ central cavity, at ∼1.9 Å, is about 0.1 Å shorter than that of porphyrins, ∼2.0 Å.) Three classes of methods may be broadly distinguished, which we dub the metal–alkyl method,^11^ the low-valent organometallic method, and the acetate/carboxylate method. The three approaches appear to be particularly suited for early, middle, and late 4d/5d transition metals, respectively. In this Account, we focus particularly on the latter two approaches, since many of the detailed procedures were worked out in our laboratory.

**Scheme 1:**
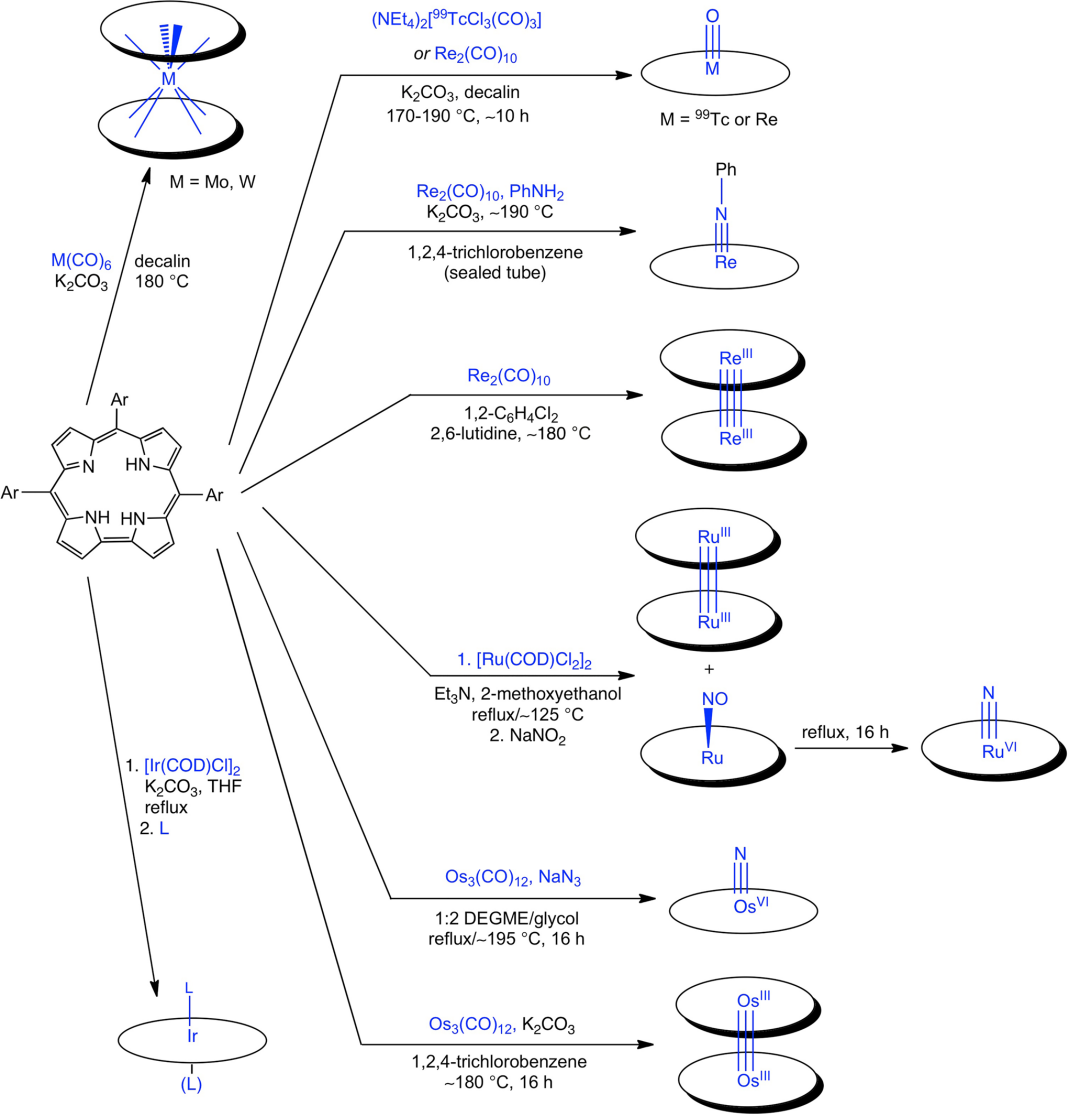
Synthetic Methods for 4d and 5d Metallocorroles

Understandably, the synthetic procedures for 5d metallocorroles tend to be capricious, with the metal source, solvent, temperature, and added base all playing decisive roles in the formation of the final product. This point may be appreciated from Scheme 1, which summarizes the protocols developed to date with the low-valent organometallic approach. For rhenium, for instance, small differences in reaction conditions favor either ReO corroles or metal–metal-bonded Re corrole dimers. While a high-boiling solvent (such as decalin, 1,2-dichlorobenzene, or 1,2,4-trichlorobenzene), Re_2_(CO)_10_, a base, and anaerobic conditions are required for both products, somewhat lower temperatures (160–180 °C) may be used for ReO corroles in contrast to Re corrole dimers, which require temperatures ≳180 °C. The key difference appears to lie in the nature of the base: potassium carbonate is the preferred base for ReO corroles and notably fails to afford Re corrole dimers; 2,6-lutidine appears to be the preferred choice for the latter. Thus, it appears that the oxygen atom of ReO corroles derives at least in part from potassium carbonate (as well as adventitious O_2_). In the same vein, homologous 4d and 5d elements such as Ru and Os, for all their qualitative similarities, typically require different experimental conditions for insertion into corroles. Overall, the 4d and 5d metallocorroles have given us broad opportunities to practice the coordination chemist’s art, opportunities that in our opinion are far from exhausted.

The new compounds emerging out of this endeavor have yielded a treasure trove of structural,^12^ spectroscopic, and electrochemical data,^13^ allowing for detailed studies of periodic trends and relativistic effects^14^ in coordination chemistry. While perusing this Account, the reader is encouraged to examine the data in Table 1 to discern structural trends and resort to the Cambridge Structural Database^15^ for additional details using the refcode provided for each structure.

**Table 1:** Selected Structural Data (Å) from Representative Transition Metal Corroles with Emphasis on 4d and 5d Metallocorroles^a^

complex	M–N_1_/N_4_^b^	M–N_2_/N_3_^b^	M–N_plane_^c^	M–L^d^	conformation	CSD refcode
**Group 4**						
Ti[Mes_2_*p*OCH_3_PC]Cl	1.993	1.988	0.667	2.220	domed	NIYDUW
{Zr[Mes_2_*p*OCH_3_PC](μ-Cl)-(THF)}_2_	2.161–2.166		1.355	2.687	domed	NIYFAE
{Hf[Mes_2_*p*OCH_3_PC](μ-Cl)}_2_	2.142–2.157		1.184	2.208	domed	NIYDEG
**Group 5**						
{Nb[Mes_2_*p*OCH_3_PC]}_2_(μ-O)_3_	2.113	2.121	0.987	2.075	domed	AQUBAS
{Ta[Mes_2_*p*OCH_3_PC]}_2_(μ-O)_3_	2.099	2.111	0.967	2.063	domed	AQOZUE
Ta[Mes_2_*p*OCH_3_PC](N^*t*^Bu)	2.085	2.046	0.775	1.778	domed	UCIVEK
Ta[Mes_2_*p*OCH_3_PC]Cl_2_	2.065	2.074	0.903	2.374	domed	UCIVAG
**Group 6**						
Cr[TPFPC](O)	1.928	1.943	0.562	1.570	planar	WIZRUS
Cr[TPFPC](NMes)	1.938	1.948	0.537	4.636	planar	NAQDAL
Mo[TPFPC](O)	2.034	2.0385	0.729	1.684	domed	YEBTIJ
Mo[T*p*OCH_3_PC]Cl_2_	2.033	2.0635	0.884	2.3697	domed	NEMMAW
Mo[T*p*CH_3_PC]_2_	2.160–2.228		1.179	–	domed	HAPVEC
{W^VI^[TPFPC]}_2_(μ-O)_3_	2.058(7)–2.124(7)		0.961	2.0105	domed	CAWVAZ
W[Mes_2_*p*OCH_3_PC]Cl_2_	2.032	2.056	0.867	2.3675	domed	WUNZUC
W[TPC]_2_	2.150–2.218		1.171		domed	OKIJID
**Group 7**						
Mn[T*p*CH_3_PC](Cl)	1.917	1.930	0.374	2.295	planar	VIHNAE
Mn[TPh_3_PC](O)	1.899	1.920	0.527	1.633	planar	NICFOX
Tc[T*p*OCH_3_PC](O)	1.981	2.002	0.687	1.660	domed	NATCAP
Re[T*p*FPC](O)	1.991	2.009	0.704	1.668	domed	NAGXEB
Re[T*p*FPC](NPh)	2.000	2.013	0.693	1.721	domed	OJEBAJ
Re[Cl_8_T*p*CH_3_PC](O)	1.9935	2.018	0.671	1.677	domed	IPUNUF
Re[Br_8_T*p*FPC](O)	1.9965	2.015	0.668	1.673	saddled/domed	IPUPAN
{Re[T*p*CH_3_PC]}_2_	2.0035	2.0145	0.531	2.236	domed	ISUREW
**Group 8**						
Fe[TDCPC](NO)	1.900	1.920	0.452	1.641	planar	AGULET
Ru[TPFPC](NO)	1.967	1.998	0.54	1.715	domed	HUQJEI
Ru[TPC](N)	1.969	1.997	0.605	1.613	domed	HAWXIP
{Ru[T*p*CF_3_PC]}_2_	1.963	1.982	0.517	2.183	domed	HAWXUB
Os[T*p*CF_3_PC](N)	1.981	1.999	0.605	1.643	domed	POPTOF
Os[Cl_8_TPC](N)	1.985	1.995	0.565	1.636	domed	QUFYAU
(AsPh_4_){Os[T*p*CF_3_TPC](N)-PtCl_3_}	1.975	1.983	0.578	1.660	domed	QUFYEY
{Os[T*p*CF_3_PC]}_2_	1.979	1.995	0.522	2.240	domed	NODDUI
**Group 9**						
Co[T*p*MePC](py)_2_	1.869	1.900		1.991	planar	FIFXEA
Co[TPFPC](PPh_3_)	1.871	1.885	0.262	2.205	planar	BAQPUF
Rh[TPFPC](py)_2_	1.942	1.966		2.066	planar	HIQDET
Rh[TPFPC](PPh_3_)	1.963	1.972	0.227	2.222	domed	MELBUA
Ir[TPFPC](Me_3_N)_2_	1.954	1.975		2.186	planar	COHYII
Ir[Br_8_TPFPC](Me_3_N)_2_	1.961	1.988		2.189	planar	COHYOO
**Group 10**						
(PyMe){Pd[TPFPC]}	1.928	1.947			planar	MUDKOO
Pt^IV^[TPC^•^](*m*-C_6_H_4_CN)(*p*-C_6_H_4_CH_3_)	1.947	1.969		2.1165	planar	FOKQAZ
Pt^IV^[TPC](*m*-C_6_H_4_CN)(py)	1.944	1.9605		2.1245	planar	IQOHII
**Group 11**						
Cu[TPC]	1.893	1.892			saddled	KAGGIJ
Cu[Br_8_T*p*MePC]	1.916	1.916			strongly saddled	FUNPIP
Cu[(CF_3_)_8_T*p*FPC]	1.926	1.924			exceptionally saddled	OVEVAN
Ag[T*p*FPC]	1.943	1.964			saddled	BAYSUR
Ag[Br_8_T*p*MePC]	1.983	1.983			strongly saddled	FUNPOV
Au[T*p*FPC]	1.939	1.956			slightly saddled	BAYSOL
Au[Br_8_TPFPC]	1.937	1.970			slightly saddled	UCEXUX
Au[Br_8_T*p*SF_5_PC]	1.937	1.963			planar	AGACAP
Au[(*p*CF_3_P)_8_TPC]	1.941	1.964			planar	NISCEA
Au[(CF_3_)_8_T*p*FPC]	1.9505	1.935			planar	LUHLUX
Au[I_4_TPFPC]	1.9145	1.935			almost planar	EBOZAZ
Au[(CF_3_)_4_TPFPC]	1.935	1.935			planar	CEBVEN

a Abbreviations: TDCPP = *meso*-tris(2,6-dichlorophenyl)corrolato; TPFPC = *meso*-tris(pentafluorophenyl)corrolato; TPC = *meso*-triphenylcorrolato; TpXPC = *meso*-tris(*p*-X-phenyl)corrolato.

b N_1_ and N_4_ refer to the two nitrogens within the bipyrrole unit of the corrole; N_2_ and N_3_ refer to the other two nitrogens.

c M–N_plane_ refers to the displacement of the M atom from the best-fit plane of the four central nitrogens.

d L refers to the axial ligand(s).

## 2 Early 4d and 5d Metallocorroles (Groups 4 and 5)

The chemistry of group 4 and 5 metallocorroles was largely developed by John Arnold and co-workers and has been reviewed by these authors.^11^ In addition, Arnold’s former student, Heather Buckley, in her Ph.D. thesis, has provided a lively first-person account of this chemistry.^16^ The syntheses generally involved some variant of the metal–alkyl method. The lithium salt of the electron-rich ligand 10-(4-methoxyphenyl)-5,15-dimesitylcorrole, Li_3_[Mes_2_*p*OMePhC]^17^ (Mes = mesityl), proved to be a particularly versatile intermediate in this context. In other cases, the use of a 4d or 5d metal–alkyl precursor allowed the researchers to sidestep the lithium salt and directly access the metallocorrole of interest. Several of the halido and imido complexes proved extraordinarily sensitive to hydrolysis, thwarting attempts at obtaining crystal structures and underscoring the consequences of the highly electropositive and oxophilic nature of these elements. While space does not permit a more detailed discussion, these complexes provide a counterpoint to the far more rugged middle and late 5d metallocorroles that are the main subject of this Account.

## 3 Group 6 Metallocorroles (Mo and W)

Molybdenum-oxo corroles are some of the most rugged among metallocorroles and are readily synthesized by heating a free-base corrole, Mo(CO)_6_, and a base such as K_2_CO_3_ in a high-boiling solvent.^18^ Oddly, the analogous WO corroles are still unknown. While we do not seriously doubt their existence, attempted insertion of W into H_3_[TPFPC] with WCl_6_ resulted in the triply bridged binuclear complex {W^VI^[TPFPC]}_2_(μ-O)_3_ (Figure 1).^19^ Because of relativistic destabilization of the 5d orbitals, WO corroles might be slightly more easily oxidized than MoO corroles. Interestingly, interaction of Li_3_[Mes_2_*p*OMePhC] with WCl_6_ in toluene affords a W^V^Cl_2_, as opposed to a W^VI^Cl_3_, corrole (Figure 1).^20^ An analogous Mo complex, Mo[T*p*OCH_3_PC]Cl_2_, has been synthesized by Bröring and co-workers via the interaction of MoO corroles and SiCl_4_.^21^ Careful examination of the published X-ray structure of the latter complex (CSD code NEMMAW) reveals a distinctive skeletal bond length alternation within the bipyrrole part of the molecule, which is often associated with a partial or full corrole radical. Unpublished DFT calculations in our laboratory also suggest a noninnocent corrole for Mo[TPC]Cl_2_ complexes. Should these conclusions be borne out with additional evidence, MoCl_2_ corroles would provide unusual examples of ligand noninnocence arising from 4d−π orbital interactions.

In our laboratory, we found that the high-temperature interaction of a free-base corrole with Mo(CO)_6_/W(CO)_6_ under strictly anaerobic conditions results in unique, eight-coordinate metallobiscorroles (Scheme 1 and Figure 1).^22,23^ Fascinatingly, the complexes are chiral, thanks to their square antiprismatic coordination, and also conformationally stable and have been successfully resolved via chiral HPLC.^24^ Their potential deployment as an inherently chiral structural element remains a fascinating prospect.

## 4 Group 7 Metallocorroles (Tc and Re)

**Figure 2:**
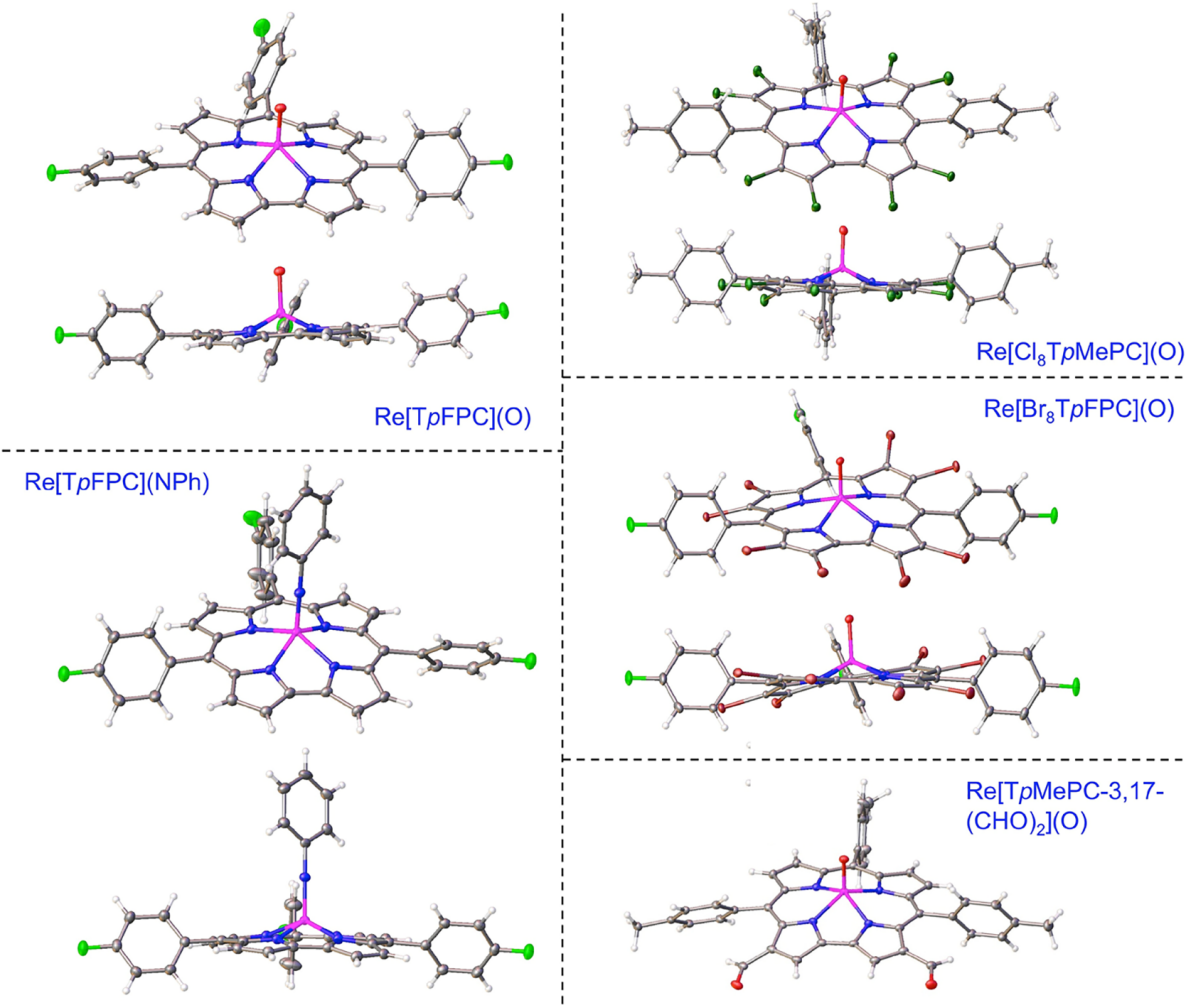
X-ray structures of selected Re corroles. See Table 1 for key structural data, including those for NAGXEB, IPUNUF, IPUPAN, and OJEBAJ.

Among all 5d metallocorroles, ReO corroles are arguably the simplest to synthesize (Scheme 1 and Figure 2). Interaction of Re_2_(CO)_10_ with free-base corroles in refluxing decalin in the presence of K_2_CO_3_ affords 50–70% yields of ReO corroles.^4^ In a variation of the method, addition of aniline to the reaction mixture resulted in good yields of Re-imido corroles.^25^ A collaboration with Roger Alberto of the University of Zurich resulted in the insertion of ^99^Tc under similar conditions with (NEt_4_)_2_[^99^TcCl_3_(CO)_3_];^26^ suitable conditions for ^99m^Tc insertion, however, have yet to be worked out.

Rhenium-oxo corroles are highly stable and aptly viewed as thermodynamic sinks of Re/corrole chemistry. They do, however, undergo electrophilic substitutions typical of aromatic compounds (see Figure 2 for X-ray structures of selected products). Exposure to elemental bromine over several days affords β-perbrominated derivatives. β-Perchlorination with elemental chlorine is much quicker, occurring over minutes.^27^ Vilsmeier–Haack formylation (with DMF/POCl_3_) also occurs smoothly, affording 3-formyl products in a highly regioselective manner.

## 5 Group 8 Metallocorroles (Ru and Os)

Both metals may be inserted into corroles via the low-valent organometallic approach (Scheme 1).

**Figure 3:**
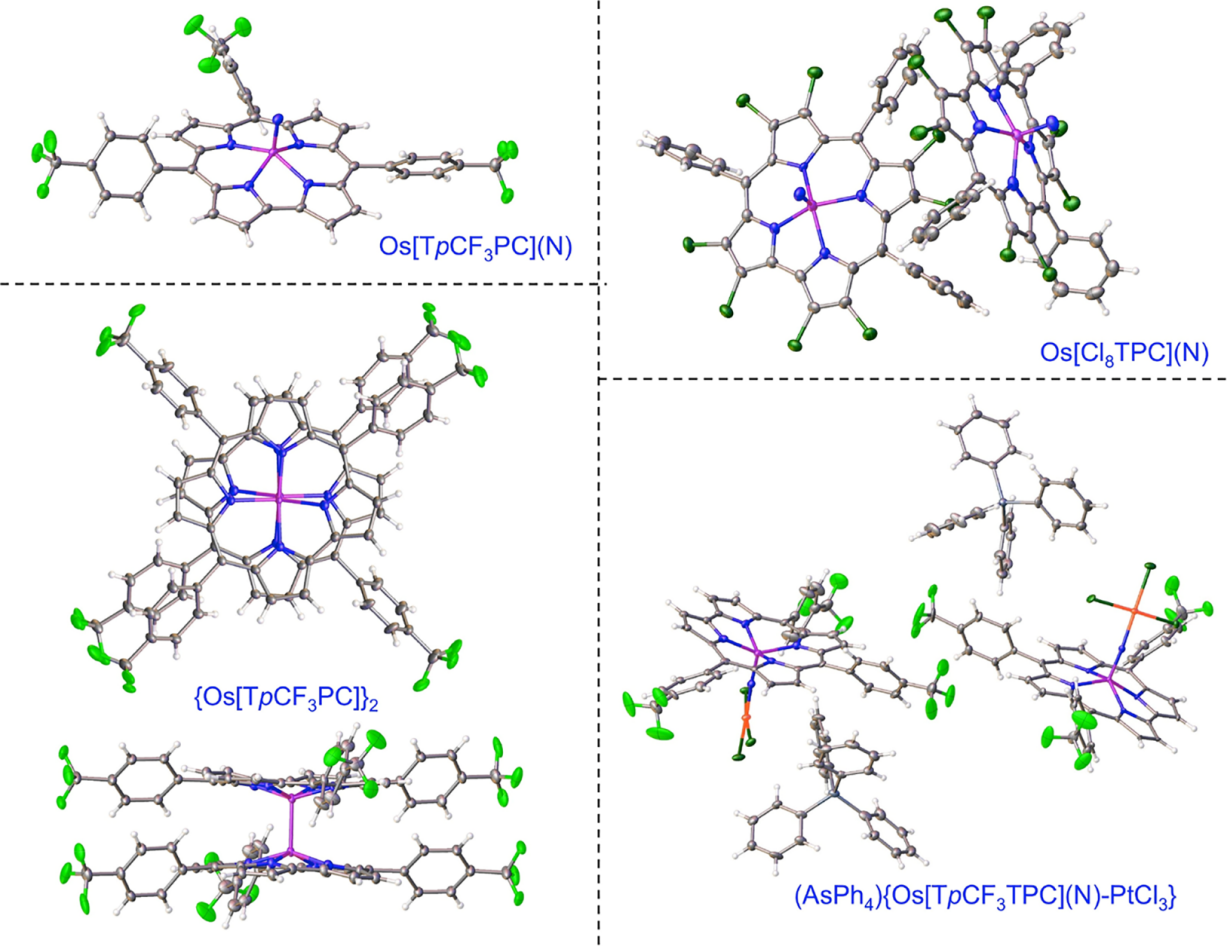
X-ray structures of selected Os corroles. See Table 1 for key structural data: POPTOF, QUFYAU, QUFYEY, and NODDUI.

Ruthenium insertion is most conveniently accomplished with [Ru(COD)Cl_2_]_*x*_ (*x* ≥ 2) in refluxing 2-methoxyethanol with triethylamine as a quencher to neutralize the liberated HCl.^28,29^ To effectively intercept the Ru corrole monomer, a trapping agent such as nitrite needs to be added within a minute or two of the beginning of the reaction. Under these conditions, the major product is a {RuNO}^6^ corrole, along with some Ru corrole dimer. Upon prolonged heating, the RuNO corrole transfers its terminal oxygen to an unknown substrate, affording Ru-nitrido corroles.^30^ For Os insertion, the reagent of choice is Os_3_(CO)_12_ and the reaction is slower and carried out at a higher temperature (∼180 °C) in 1:2 DEGME/ethylene glycol [DEGME = 2-(2-methoxyethoxy)ethanol] with NaN_3_ as a trapping agent for the initially formed Os corrole monomer.^3^ The resulting OsN corroles, like their ReO counterparts,^4^ are exceptionally stable, but also amenable to electrophilic aromatic substitution. Thus, β-octachlorination has been accomplished with elemental chlorine, and an OsN octachlorocorrole has been structurally characterized (Table 1 and Figure 3; CSD code QUFYAU). In an attempt to elicit more interesting reactivity, the photochemical interaction of an OsN corrole with Zeise’s salt resulted in a binuclear an Os^VI^≡N–Pt^II^ complex. The very short OsN–Pt linkage [1.895(9)–1.917(8) Å] (Table 1 and Figure 3; CSD code QUFYEY) and the downfield ^195^Pt NMR resonance (−2702 ppm) strongly indicated that the OsN corrole acts as a π-accepting ligand toward the Pt(II) center.^31^ The reaction provides a rare example of the successful photochemical activation of a metal–ligand multiple bond that is too kinetically inert to exhibit any significant reactivity under thermal conditions.

## 6 An Interlude on Metal–Metal Multiple Bonding

**Figure 4:**
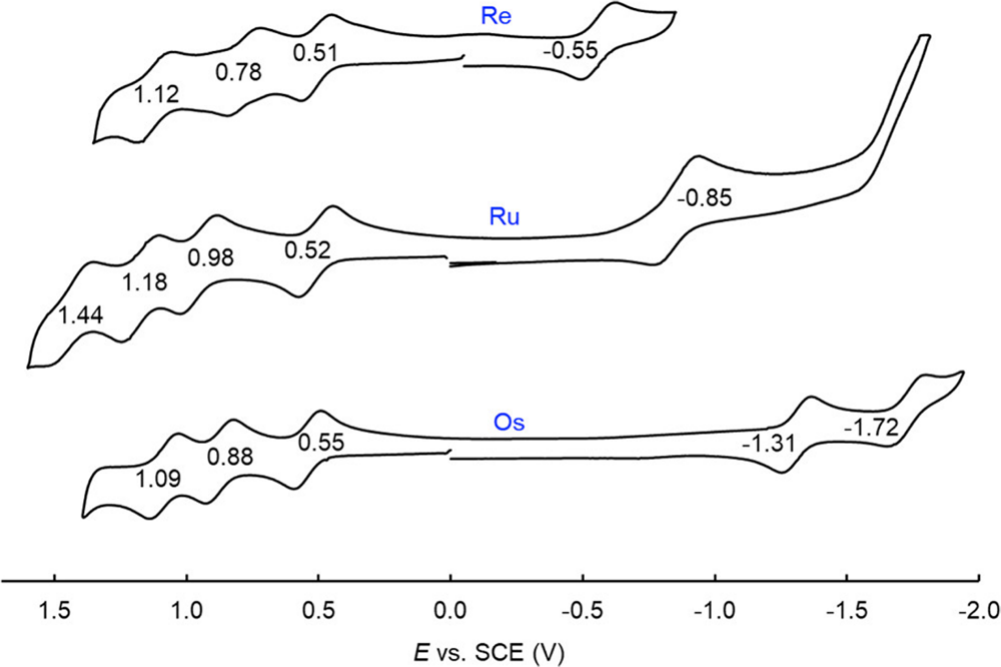
Comparative cyclic voltammograms for {M[T*p*MePC]}_2_, where M = Re, Ru, and Os. Reproduced with permission from ref (33). Copyright 2021 American Chemical Society.

Ruthenium corrole dimers have for some time been known as dead-end products of Ru insertion into corroles.^28−30^ Two new classes of metal–metal multiple-bonded corrole dimers have recently been prepared in our laboratory, the Os^32^ and Re^33^ corrole dimers (Scheme 1). The Ru and Os corrole dimers are thought to be triple-bonded with a σ^2^π^4^δ^2^δ*^2^ bonding scheme, while the Re corrole dimers are thought to be quadruple-bonded with a σ^2^π^4^δ^2^ bonding scheme.^34^ The metal–metal bond distances are all around 2.23 ± 0.01 Å across the three families of complexes (Table 1 and Figure 3); the UV–vis spectra, to a first approximation, are also rather similar. The redox potentials on the other hand underscore dramatic electronic differences among the three compounds (Figure 4). While the oxidation potentials are similar (suggesting ligand-centered oxidation), the reduction potentials and electrochemical HOMO–LUMO gap vary dramatically as a function of the element. From Ru corrole dimers to Os corrole dimers, the reduction potential downshifts by ∼450 mV, reflecting relativistic destabilization of the metal–metal π*-based LUMO.^32^ Indeed, as a result of the high energy of this MO, some of the unpaired electron density in the Os dimer anion is thought to leak onto the corrole. In the case of an Re corrole dimer on the other hand, reduction entails electron addition to a much lower-energy δ* MO, resulting in a much less negative reduction potential. The electrochemical HOMO–LUMO gaps thus span a range of ∼800 mV across the three compounds, with {Os[T*p*MePC]}_2_ (1.86 V) > {Ru[T*p*MePC]}_2_ (1.37 V) > {Re[T*p*MePC]}_2_ (1.06 V) (Figure 4).^33^

## 7 Group 9 Metallocorroles (Ir)

As mentioned above, Ir was the first 5d transition metal that was deliberately inserted into a corrole. The feat was accomplished by Joshua Palmer, then a Ph.D. student of Harry Gray’s at Caltech.^8^ The Caltech researchers and Zeev Gross were inspired by a then-recent report of water oxidation by Mes_3_Ir^V^O and surmised that corrole might stabilize a similar high-valent Ir center.^35^ Although Ir was duly inserted (Scheme 1), the desired high-valent chemistry failed to materialize. A large number of six-coordinate 18-electron Ir corrole bis-amine adducts have been synthesized, as well as a smaller number of 16-electron monophosphine adducts (Table 1).^36,37^ Very recently, the use of water-soluble phosphines has provided convenient access to water-soluble, five-coordinate Ir corroles.^38^

## 8 Group 10 Metallocorroles (Pd and Pt)

**Figure 5:**
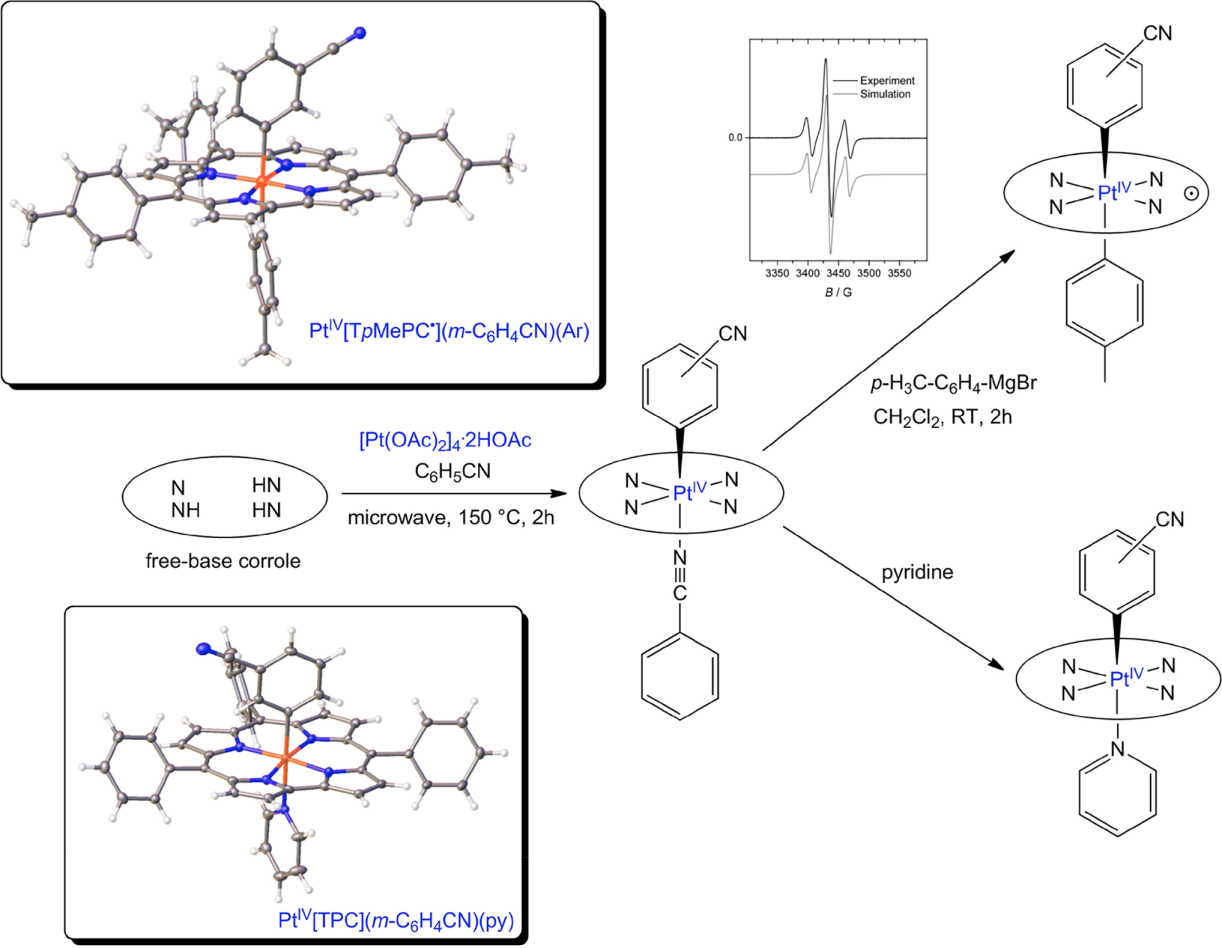
A snapshot of current Pt corrole chemistry: key reaction pathways and X-ray structures. See Table 1 for key structural data: FOKQAZ and IQOHII.

A good way of describing synthetic methods currently available for group 10 corrole derivatives is to say that there is significant scope for improvement. A handful of key breakthroughs have taken place, however. Palladium(II) was inserted in H_3_[TPFPC] via interaction with Pd(OAc)_2_ in pyridine; the {Pd[TPFPC]}^−^ anion was finally isolated in stable form as the *N*-methylpyridinium salt.^39^ No Pd(IV) corroles have been reported. Platinum(IV) corroles, on the other hand, have been synthesized in our laboratory; the protocols, however, are quite unsatisfactory.^2^ A wide range of Pt sources and a variety of reaction conditions were explored, all without success. Ultimately, through pure serendipity, the interaction of free-base corroles with (*commercially unavailable!*) tetranuclear platinum acetate, [Pt(OAc)_2_]_4_·2HOAc, in benzonitrile at 140–150 °C under aerobic conditions and microwave irradiation was found to afford low yields (∼6%) of diamagnetic, six-coordinate Pt^IV^Ar corroles; note that the axial Ar group is derived via C–H activation of PhCN (Figure 5). These products, however, proved unstable.^2^ Attempted displacement of the PhCN-*N* ligand with Ar′MgBr led to neutral, paramagnetic Pt[T*p*XPC](Ar)(Ar′) derivatives, a few of which yielded single-crystal X-ray structures. Several lines of evidence, including EPR spectroscopy, established that these complexes are full-fledged metalloradicals: Pt^IV^[T*p*XPC^•2–^](Ar)(Ar′). The unstable PhCN-*N* complexes were also found to react with pyridine, affording stable Pt(IV) complexes with the general formula Pt^IV^[T*p*XPC](Ar)(py) (Figure 5).^40^ All the structurally characterized products showed strictly planar Pt-corrole cores with short Pt–N distances of 1.957 ± 0.012 Å (Table 1).

## 9 Group 11 Metallocorroles

The chemistry of Au corroles had somewhat of a rough start. Attempted Au insertion into simple corroles with AuCl_3_ or KAuCl_4_ failed, as the oxidation-prone macrocycles broke apart under the influence of the highly oxidizing Au reagents. With the then-newly available free-base β-octabromo-*meso*-triarylcorroles,^41,42^ Au insertion finally worked, an important early step in the historical development of the 5d metallocorrole field.^9,10^ Unfortunately, the Au octabromocorrole products proved poorly soluble and resistant to yielding X-ray quality crystals.^9,10^

**Figure 6:**
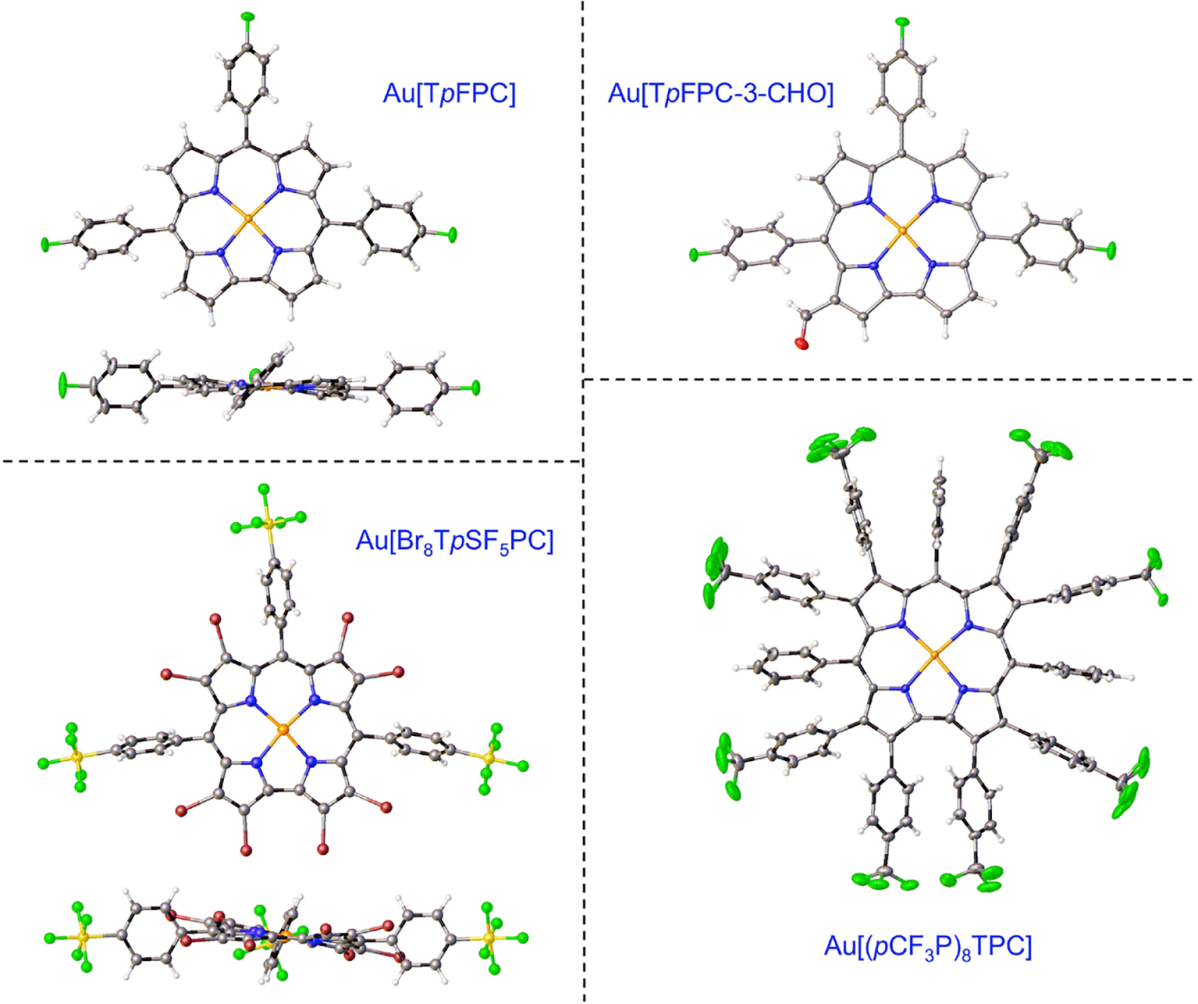
X-ray structures of selected Au corroles. See Table 1 for key structural data, including BAYSOL, AGACAP, and LUHLUX.

The use of Au(III) acetate finally provided a reliable method for Au insertion into simple *meso*-triarylcorroles, underscoring the power of the “acetate method”.^1^ The acetate method is still the method of choice today, affording Au corroles for a variety of applications. Even for β-octabromocorroles, the acetate method has become the method of choice. The synthesis of Au[Br_8_T*p*SF_5_PC] (Figure 6)^43^ via the acetate method from the corresponding free-base corrole established that fluorinated substituents such as SF_5_ help solubilize Au octabromocorroles. In another study, interaction of an undecaarylisocorrole with Au acetate resulted in aromatization, affording a gold undecaarylcorrole (Figure 6).^44^ Finally, the Au-acetate method has even worked for azulicorrole, a rare example of a carbacorrole.^45^

Like their ReO counterparts, Au triarylcorroles undergo Vilsmeier–Haack formylation but afford symmetric 3,17-diformyl derivatives. The preference for diformylation appears to be related to the somewhat lower oxidation potentials (and hence greater nucleophilicity) of Au corroles, relative to ReO corroles. Note that Figures 2 and 6 depict as yet unpublished minor products, a ReO 3,17-diformylcorrole and a Au 3-formylcorrole, since these are the compounds that yielded crystal structures.

**Figure 7:**
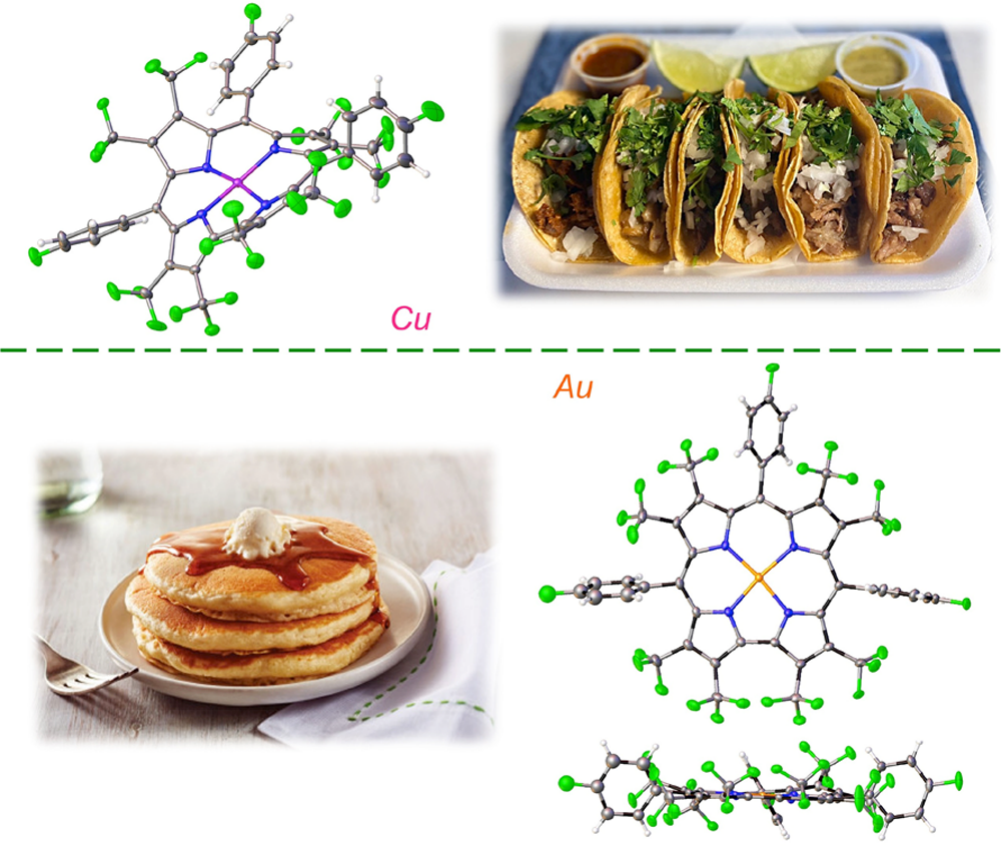
X-ray structures of Cu[(CF_3_)_8_T*p*FPC] (the “taco”) and Au[(CF_3_)_8_T*p*FPC] (the “pancake”).

Gold corroles provide paradigmatic examples of planar (or slightly saddled) four-coordinate metallocorroles.^1,43,44^ In so doing, they offer a sharp contrast to Cu corroles, which are *inherently* saddled. Saddling in the Cu case switches on a Cu(d_*x*^2^–*y*^2^_)–corrole(π) orbital interaction, allowing some of the corrole π-electron density to flow into the space of the Cu(d_*x*^2^–*y*^2^_) orbital.^46−54^ Copper corroles are accordingly rightly viewed as having substantial Cu^II^-corrole^•2–^ character. In the case of Au corroles, the relativistically destabilized 5d_*x*^2^–*y*^2^_ orbital is far too high in energy to effectively overlap with the corrole’s π-HOMO, which accounts for the planarity of Au corroles. One of the most dramatic illustrations of the difference in conformational preference between Cu and Au corroles is provided by the isoelectronic β-octakis(trifluoromethyl)-*meso*-triarylcorrole complexes, M[(CF_3_)_8_T*p*FPC] (M = Cu, Au; Figure 7): while the Cu complex is folded like a taco (CSD code OVEVAN),^55^ with adjacent pyrrole rings tilted by as much as 85° relative to each other, the Au complex is flat as a pancake (CSD code LUHLUX).^56^

## 10 Photophysical Properties and Applications As Phototherapeutics

**Figure 8:**
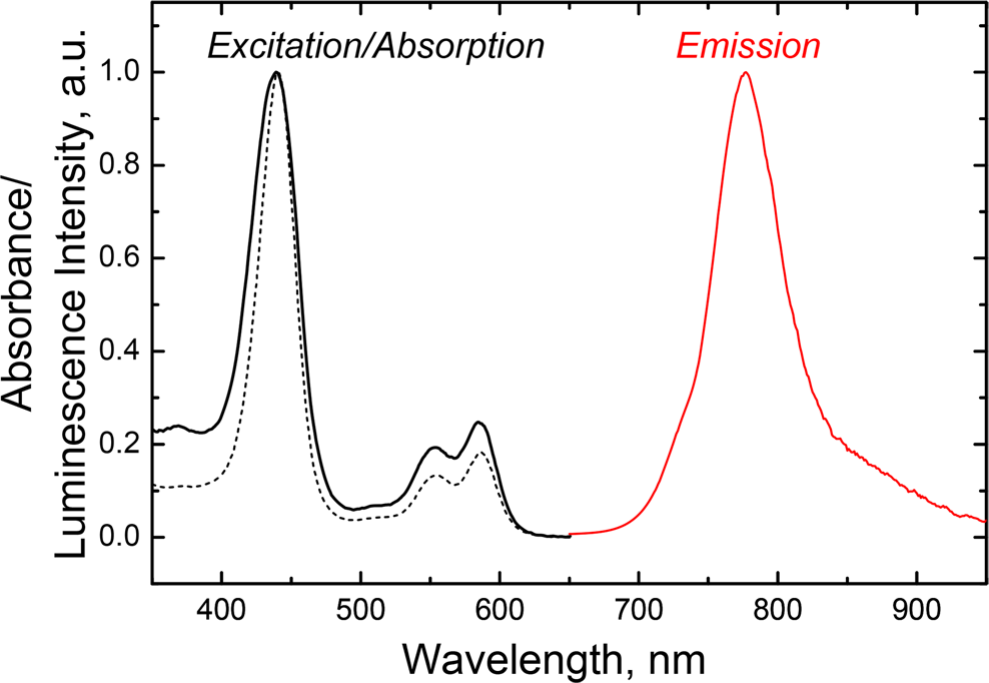
Absorption (black dashed line), phosphorescence excitation (black solid line; λ_em_ 775 nm) and emission (red line; λ_ex_ 590 nm) spectra of Re[T*p*CF_3_PC](O) in toluene. Phosphorescence spectra were measured under anoxic conditions.

The middle and late 5d metallocorroles, involving the elements Re–Au, exhibit room-temperature phosphorescence in the near-infrared (Table 2 and Figure 8).^57^ Although the phosphorescence quantum yields range from low to moderate (Ir^37,58^ < Au^59^ < Pt(IV)^40^ < OsN^60^ < ReO^61^), the complexes in general efficiently sensitize singlet oxygen formation, promising applications as sensitizers in photodynamic therapy. Indeed, amphiphilic Au and ReO triarylcorroles have already exhibited impressive *in vitro* photocytotoxicity against multiple cancer cell lines.^59,62^ We remain excited about the possibility of developing nanoconjugated platforms based on 5d metallocorroles, with improved tumor-targeting and imaging (theranostic) capabilities.

**Table 2:** Photophysical Properties of Representative 5d Metallocorroles in Anoxic Toluene at 23 °C^a^

	absorbance maxima (nm)						
compound	Soret	Q	Q	emission maximum (nm)	lifetime (μs)	rel. quantum yield (%)^b^	ref
Re[T*p*CF_3_PC](O)	440	553	586	777	74	1.52	(61)
Os[T*p*CF_3_PC](N)	444	553	593	779	183	0.39	(60)
Ir[TpCF_3_PC)]py_2_	416	602		836	5.6	∼0.04	(37)
Pt^IV^[T*p*CF_3_PC](*m*-C_6_H_4_CN)(py)	430	569	595	813	23	0.27	(40)
Au[T*p*CF_3_PC]	424	564	576	788	98	0.19^c^	(59)
Pt[TPTBP]^b^	430	564	614	770	48	21	(63)

a λ_ex_ = 560–614 nm (at approximately the Q-band maximum).

b Unless otherwise mentioned, Pt(II) tetraphenyltetrabenzoporpyhrin (Pt[TPTBP]),^63^ was used as the reference for quantum yield measurements.

c This quantum yield is relative to that for fluorescence of rhodamine 6G in ethanol.

Other potential applications have also been briefly examined. Thus, optical sensors based on OsN^60^ and ReO^61^ corroles exhibit excellent photostability and sensitize singlet oxygen formation with quantum yields >0.75. The complexes have also been found to be promising sensitizers in triplet–triplet annihilation-based upconversion systems. Interestingly, a Au corrole with carboxylic acid anchoring groups has been found to exhibit a surprisingly high power conversion efficiency of 4.2% in dye-sensitized solar cells. The observed photovoltaic activity was initially attributed to triplet-state reactivity of the Au corrole.^59^ Subsequently, it became clear that the T_1_ state is simply not energetic enough to inject an electron into titania. Femtosecond time-resolved transient absorption measurements also strongly indicated that photovoltaic activity reflects electron injection for the S_1_ state, which effectively competes with intersystem crossing.^64^ Interestingly, ReO and OsN corroles exhibit much poorer photovoltaic activity, possibly as a result of shorter S_1_ lifetimes.

## 11 Concluding Remarks

What began a few years ago as an amusing exercise to create misfit metal–ligand assemblies has by now yielded a dozen or so families of 5d metallocorroles and a wealth of insights into fundamental questions of structure and bonding and new building blocks for photomedicine.

The low-valent organometallic method has arguably proved the most versatile, affording chiral Mo and W biscorroles, metal–metal-bonded dimers, and a variety of 1:1 metal–corrole derivatives. The acetate method provided a practical route to Au corroles and, less satisfactorily, to platinum(IV) corroles. Gratifyingly, the great majority of middle and late 5d corrole derivatives have proved thermally and photochemically rugged, as well as amenable to selective electrophilic aromatic substitution, foreshadowing a variety of practical applications.

Several of the middle and late 5d corrole derivatives have been found to exhibit NIR phosphorescence under ambient conditions. Although the phosphorescence quantum yields are low (Ir) to moderate (ReO, OsN, Pt, and Au), the complexes have all been found to efficiently sensitize singlet oxygen formation. In addition, amphiphilic Au and ReO corroles have been found to exhibit impressive photocytotoxicity against multiple cancer cell lines *in vitro*. We thus envision a bright future for biomedical applications of the 5d metallocorroles.^65^ Bio-, nano-, and radioconjugation promise a wide range of multimodal therapeutic and theranostic agents for cancer. Photothermal therapy, potentially involving metallocorrole nanoconjugates, and combined photodynamic–photothermal therapy, in particular, remain fascinating prospects.

Some of the Au corroles have also proved effective as photosensitizers in dye-sensitized solar cells. Although this photovoltaic activity was originally ascribed to triplet state reactivity, it has become clear that the triplet state is not energetic enough to inject electrons into titania. Instead, femtosecond transient absorption spectroscopy strongly suggests that it is the excited singlet state that does the electron injection, effectively competing with intersystem crossing.

Finally, it is worth emphasizing that opportunities for creative coordination chemistry are far from exhausted. The development of a higher-yielding route to Pt(IV) is an obvious gap in our current knowledge. In the same vein, key aspects of axial coordination chemistry, involving carbene, carbyne, and carbon-atom (carbide) ligands, remain to be uncovered for W, Re, Os, and Ir corroles. Crazier targets include superhigh-valent species such as heptavalent rhenium or iridium or octavalent osmium complexes with nitride, carbide and other, related axial ligands.^66^ We remain optimistic that some “misfit” chemist, somewhere, will successfully synthesize one or more of these species.
